# Supplementary material for: Mutations in PCYT2 disrupt etherlipid biosynthesis and cause a complex hereditary spastic paraplegia
Source: Brain. 2019 Oct 22;142(11):3382–97. doi: 10.1093/brain/awz291 (PMC6821184; doi:10.1093/brain/awz291)
Supplement: awz291_Supplementary_Data [file awz291_supplementary_data.zip › awz291-Suppl_data/Supplementary_Data4.zip!/Supplementary information 4 - plasma lipidomics.zip!/Plasma lipidomics/Control vs Patient/Statistical plots and data/DG species/Volcano plot_alternative.pdf]

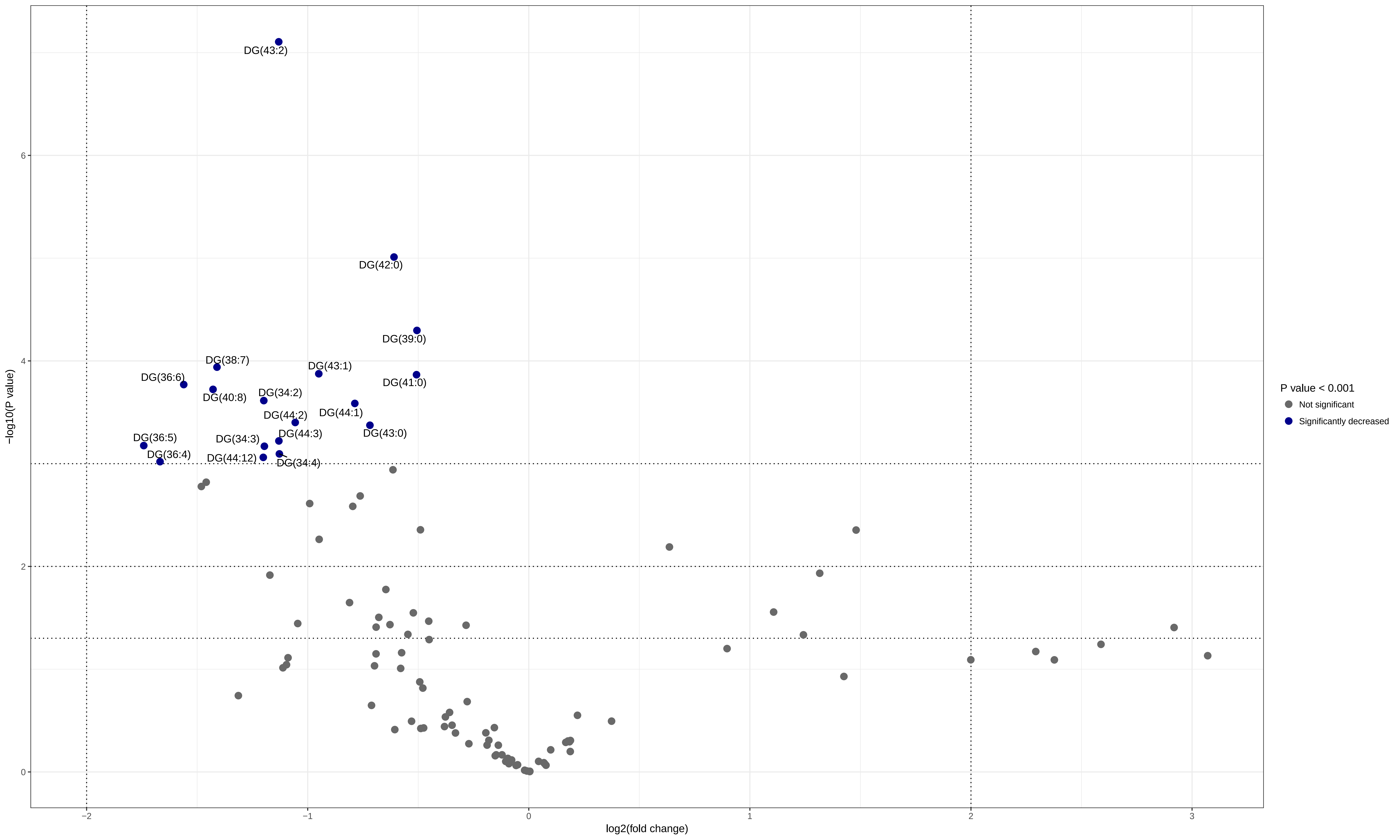

DG(43:2)
DG(42:0)
DG(39:0)
DG(38:7)
DG(43:1)
DG(36:6)
DG(41:0)
DG(40:8)
DG(34:2)
DG(44:1)
DG(44:2)
DG(43:0)
DG(36:5)
DG(34:3)
DG(44:3)
DG(36:4)
DG(44:12)
DG(34:4)
−log10(P value)
log2(fold change)
P value < 0.001
Not significant
Significantly decreased
